# Modification of photocatalytic activity and antibacterial properties of Mn_2_O_3_ by Zn ions doping

**DOI:** 10.1038/s41598-025-95769-2

**Published:** 2025-04-24

**Authors:** Rabab A. Nasr, Ahmed F. El‑Sayed, G. M. El Komy, Gehan T. El‑Bassyouni, Sahar M. Mousa

**Affiliations:** 1 Water Pollution Research Department, Environment and Climate Changes Research Institute, National Research Centre, 33 El Bohouth St., Dokki, Giza, 12622 Egypt; 2 Microbial Genetics Department, Biotechnology Research Institute, National Research Centre, 33 El Bohouth St., P.O. 12622, Cairo, Egypt; 3 Egypt Center for Research and Regenerative Medicine (ECRRM), Cairo, Egypt; 4 Molecular Modeling and Spectroscopy Laboratory, Centre of Excellence for Advanced Science, National Research Centre, Gizza, Egypt; 5 Electron Microscope and Thin Films Department, Physics Research Institute, National Research Centre, 33 El-Buhouth St, P.O. 12622, Dokki, Cairo Egypt; 6 Refractories, Ceramics and Building Materials Department, Advanced Materials Technology and Mineral Resources Research Institute, National Research Centre, 33 EL-Buhouth St, P.O. 12622, Dokki, Cairo Egypt; 7 Inorganic Chemistry Department, Advanced Materials Technology and Mineral Resources Research Institute, National Research Centre, 33 EL-Buhouth St, P.O, Dokki, 12622 Cairo Egypt

**Keywords:** Zinc-doped Mn_2_O_3_ NPs, Photodegradation, Photoluminescence, Methyl green, Antibacterial activity, Environmental sciences, Chemistry, Materials science

## Abstract

This study focuses on the fabrication of zinc-doped manganese oxide (Mn_2_O_3_) nanoparticles (NPs) to unleash their potential as high-performance photocatalysts. Zn-doped Mn_2_O_3_ nanoparticles (NPs) were prepared via a precipitation method, with fine adjustment of Zn content (3%, 5%, and 10%). The structural evolution from cubic Mn_2_O_3_ to tetragonal ZnMn_2_O_4_ was confirmed by X-ray diffraction (XRD). Energy dispersive X-ray (EDX) analysis confirmed the smooth Zn incorporation, while transmission electron microscopy (TEM) and FESEM revealed the transformative effect of Zn on the particle size and shape. Optical characterizations showed impressive results:UV-Vis DRS revealed a significant reduction in the band gap from 2.26 eV to 1.89 eV, enhancing light absorption. Meanwhile, the photoluminescence (PL) spectra showed vibrant emission peaks at 425, 466, 563, and 623 nm, with the intensity increasing along with the zinc content. The optical prowess of these nanoparticles was validated by the nearly complete degradation of methyl green (MG) dye under visible light. Also, Zn-doped Mn_2_O_3_ samples were evaluated against harmful pathogens such as S. aureus, E. faecalis, K. pneumoniae, P. aeruginosa, E. coli, and C. albicans.

## Introduction

Industrial organic dye runoff into water streams affects the ecosystem. These textile, paper, and pharmaceutical compounds contaminate water and endanger humans^1^ The presence of this pollutant in the water stream, limits photosynthesis, and modifies pH, impacting aquatic life and biodiversity. Antibacterial infections endanger health, healthcare, the economy, and well-being. Antibacterial infections can cause mild skin disease to fatal sepsis^2^. A quick solution to this situation is crucial. Water impurities can be removed using advanced oxidation processes (AOP), specifically photocatalysis^3^ Photocatalysis has high oxidation potential and antibacterial characteristics and at the same time has many advantages over conventional wastewater treatment, including complete mineralization producing non-toxic byproducts like water and CO_2_, cost-effectiveness using cheaper visible light or natural sunlight, and sustainable technology since no sludge or chemicals are needed^1,2^.This study investigates doping to improve the visible light efficiency of manganese oxide.

MnO, Mn_2_O_3_, and Mn_3_O_4_ are the most well-known manganese oxides, with numerous uses in catalysis and battery technology^4^. Manganese(III) oxide has the chemical formula Mn_2_O_3_ and a molecular weight of 157.88 and has two forms: α-Mn_2_O_3_ and γ-Mn_2_O_3_. Many preparations of nano-crystalline Mn_2_O_3_ have been reported, including syntheses utilizing the oxidation of Mn(II) salts or the reduction of MnO_2_^5^. Manganese oxides, specifically MnO_2_, Mn_2_O_3_, and Mn_3_O_4_, have garnered significant scientific interest for various applications, including gas sensing, batteries, supercapacitors, and magnetism^6,7^. Given that it is non-toxic, affordable, has good structural flexibility, and is compatible with the environment, Mn_2_O_3_, a p-type semiconducting material, is seen to be a likely choice^8^. Mn_2_O_3_’s application in the optoelectronic field and the potential for improving its optical characteristics is the main research focus.

The uncertain difference in ionic radii between Mn^3+^ (0.078 nm) and Zn^2+^ (0.074 nm) allows for easy integration into the Mn_2_O_3_ lattice^9,10^. Efficient dopants should not alter the structure of the host atom during incorporation^11,12^. Doping of the materials can incur various physiochemical features of the parent material, making it feasible to modify the material to achieve the desired physical and chemical qualities^13,14^. Doping with low valence positive ions disrupts the expected symmetry of metal oxides containing substituted atoms, leading to changes in electronic mobility^15–19^. ZnO is the most common photocatalytic material discovered in the literature^20^. ZnO is gaining popularity because of its efficiency, low cost, non-toxicity, and chemical and mechanical stability^21^. Although ZnO has intense photocatalytic activity, its practical application in industry is not viable owing to its broad bandgap energy (3.37 eV) and high excitation binding energy (60 meV)^22–26^. To address this constraint, ZnO is doped with transition metal oxides, which shifts light activity from the UV to the visible range by absorbing photons with lower energies as Mn_2_O_3_ that have piqued the interest of researchers as a result of its non-toxicity, high chemical, and physical stability, low bandgap energy (1.290–1.48 eV), and notable structural flexibility^27^. Recent studies have explored various materials and methods to enhance the photocatalytic and antibacterial properties where most of them focused on the Mn-doped zinc oxide and its different applications^28–31^ However, few studies were done on the Zn-doped manganese oxide^32,33^. The innovation of the current work is developing an advanced photo-catalytic material with potent antibacterial activities aiming to optimize these dual-functional systems for various applications such as water purification where. This study takes a unique approach, aiming to synthesize and evaluate the properties of both pure and zinc-doped manganese oxide NPs. The structural, morphological, photocatalytic activity, photoluminescence properties, and optical features of the produced samples were all thoroughly investigated. The antibacterial assays were conducted to determine the bioactivity of the produced samples.

## Experimental

### Preparation of Mn_2_O_3_ and doped samples with Zn

For pure manganese oxide preparation by precipitation method, 19.8 g of Manganese chloride (MgCl_2_.4H_2_O – BHD Laborator, Suppliers, 99%) was dissolved in 500mL of refined water to form (0.2 M) solution followed by dropwise addition of NaOH (2 M) solution under stirring at room temperature until the pH value of the solution equal to 10. The solid precipitation becomes brown in a few seconds. After two hours of continuous chemical reaction, the precipitate settled for the night and was repeatedly washed with distilled water to remove the surplus NaOH. This was followed by four hours of filtration and drying at 100^o^C and two hours of calcining at 500^o^C. Manganese oxide doped with Zn samples was prepared similarly to the abovementioned manganese oxide. The determined amounts of zinc acetate were added to the manganese chloride solution, resulting in a Zn/(Zn + Mn) atomic ratio (referred to as x Zn) that ranged from 0.03, 0.05, and 0.10. The subsequent procedures for creating pure manganese oxide were the same as those outlined previously. The code name of the prepared samples is shown in Table 1.

**Table 1:** The code name of prepared samples.

Code name	Meaning
Mn_2_O_3_	Pure Mn_2_O_3_ without doping
3%Zn Mn_2_O_3_	Mn_2_O_3_ doped with 3% Zn
5%Zn Mn_2_O_3_	Mn_2_O_3_ doped with 5% Zn
10%Zn Mn_2_O_3_	Mn_2_O_3_ doped with 10% Zn

### Photocatalytic activity

#### Chemicals

Methyl green (MG) is from LOBA in India, and sodium hydroxide (NaOH) and sulfuric acid are from Sigma Aldrich in Germany.

#### Photocatalytic experiments

The investigated samples (pure Mn_2_O_3_ and doped samples with Zn) and Methyl green (MG) dye were subjected to visible light via a 200-watt xenon lamp with a 400 to 800 nm wavelength range in batch operating mode. An approximate distance of 20 centimeters exists between the solution’s surface and the light. In every investigation of photocatalytic degradation, a dye solution with a concentration of 20 mg/L was used. After over 30 min of vigorous agitation, the investigated samples and MG solutions should have achieved a state of balance for the adsorption of the catalyst onto the dye surface. At regular intervals throughout irradiation, 2 milliliters of the reaction mixture were extracted, and the dye solutions were subjected to centrifugal straining to separate the catalyst particles. We measure the supernatant concentration of the BG dye solution. Using a UV-visible Jasco V630 spectrophotometer, the percentage of dye removal is ultimately determined. To examine the influence of experimental conditions on the breakdown rate of MG, a photocatalytic experiment was conducted at different pH levels (3–7), catalyst dosages (0.75–2.0 g/L), and starting concentrations (20–50 mg/L).

Five sequential experiments were carried out under optimal conditions to assess the stability of the 10% ZnMn_2_O_3_ photocatalyst using fresh MG solutions. Between tests, the photocatalyst was separated by filtration, washed away with distilled water, and dehydrated at 110 °C for 12 h.

MG removal percentage was determined using the following Eq. 

1 $$\:\text{\%}\:\text{R}\text{e}\text{m}\text{o}\text{v}\text{a}\text{l}=\frac{(C_{0}-C)}{C_{0}}\times\:100$$

Define the concentrations in mg/L of the MG before and after degradation as C_0_ and C.

### Assessment of the antimicrobial activity

All strains were obtained from the Microbial Genetics Lab., National Research Centre, Egypt. The antibacterial activity of samples against gram-positive (*S. aureus* & *Enterococcus faecalis*) and gram-negative (*E. coli*,* P. aeruginosa*) bacteria was determined by the agar diffusion method. Nutrient agar medium was prepared by adding peptone (5.0 g), yeast extract (2.0 g), NaCl (5.0 g), and agar (20 g) in one-liter water. The pH was adjusted to 7.0 and sterilized at 130 °C for 40 min in an autoclave. Following solidification, the agar nutrient medium was moved to the Muller Hinton agar plate, where the bacterial lawn was cultivated. After adding 20 mg of (Mn_2_O_3_, 3% Zn-Mn_2_O_3_, 5% Zn-Mn_2_O_3_, and 10% Zn-Mn_2_O_3_), the mixture was incubated for 24 h at 37 °C. It was determined that the bacterial growth inhibition zone was measured^34^.

### Characterization

The powdered X-ray diffraction pattern (P-XRD) of type BRUKER, D8 ADVANCED was used to examine the crystalline phases of powdered materials before and after Zn doping, Cu target, Germany, fitted with an X-ray tube and CuKα radiations (λ = 1.5406 Å) via Ni filter was employed. The diffractometer was adjusted at 40 kV, 40 mA, with a scan speed of 2° per min. The data was taken between 2θ = 20–70°. The JCPDS standard was used to identify the phases that were achieved. The Debye-Scherer equation was utilized to determine crystallite size (Cs). The structural and compositional properties of powdered materials were analyzed using FT-IR at room temperature. The study used an FT-IR spectrometer (Type A FTIR-6100, JASCO, USA) with a vibrational wave number range of 500–2500 cm^− 1^ and a spectral resolution of 4 cm^− 1^. Before qualitative analysis, powdered materials were combined with potassium bromide (KBr) at a 1:100 ratio. The mixture was compressed into discs with a 5 tons/cm^2^ load in an evocable die. The microstructure of prepared samples was investigated by using SEM (JEOL JXA-840 A, Electron probe micro-analyzer, Japan) at 15 kV. The transmission electron microscope (TEM) model JEM-2100 from Joel in Japan was used to study nanoparticles’ nature and crystallinity. Particles were drop-cast into an aqueous dispersion on a carbon-coated copper grid, air-dried at room temperature, and examined under a microscope. The optical measurements of synthesized nanoparticles were performed using a UV-visible spectrophotometer type. JASCO 570 was used to analyze the optical properties. The measurements were investigated in the wavelength range 290–2500 nm. The photoluminescence property of prepared samples was examined by a luminescence spectrophotometer (Jasco FP-6500) at 339 nm as excitation wavelength for PL measurement.

## Results and discussion

### X‑ray diffraction analysis

**Fig. 1:**
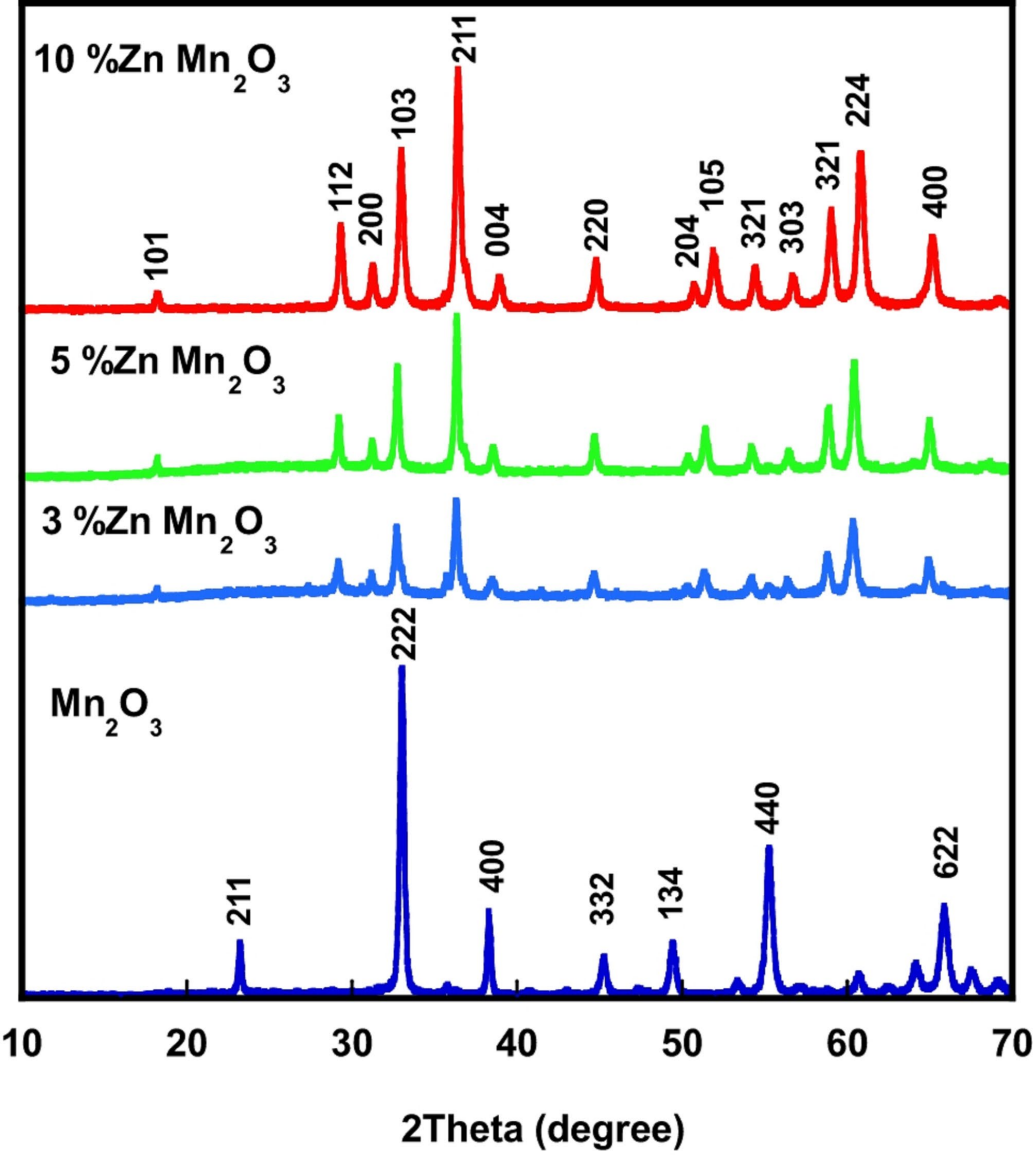
XRD patterns of pure manganese oxide NPs and doped samples with Zn (3%, 5%, and 10%).

The XRD patterns obtained for pure manganese oxide nanoparticles and doped samples with Zn at different concentrations (3, 5, and 10%) are illustrated in Fig. 1. For pure Mn_2_O_3_, the resultant peaks with respective planes are related to manganese oxide Mn_2_O_3_ phase with cubic structure [JCPDS (089-4836)] without the presence of any impurities. For Zn-doped manganese oxide samples, all obtained diffraction peaks and their respective planes are identical to ZnMn_2_O_4_ [ JCPDS card (24-1133)], where the doping of manganese oxide by Zn changes the phase structure from Mn_2_O_3_ (cubic) to ZnMn_2_O_4_ (tetragonal structure). In addition, the reflection peak intensity increases as the doping concentration of Zn is increased due to the higher reflectance of Zn ions than Mn ions. The crystallite size of pure manganese oxide nanoparticles and doped samples with Zn (3, 5, and 10%) is 15, 11,10, and 9 nm, respectively **(**Table 2). To study the effect of Zn doping on the lattice parameters of the structure of doped samples, the microstrain was estimated from XRD patterns through the following equation;

2 $$\beta \:cos\;\theta = \frac{{k\lambda }}{D} + 4\varepsilon \;sin\;\theta$$

Where, $\:\beta\:$ is the FWHM in radians and the microstrain ℇ is given by the slope of plot β cosθ vs. 4 sinθ.

**Table 2:** The crystallite size and the microstrain of pure manganese oxide NPs and doped samples with Zn (3%, 5%, and 10%).

Sample	Crystallite size (nm)	Micro strain
Pure Mn_2_O_3_	15.95	0.00037
3%Zn Mn_2_O_3_	11.03	0.00237
5%Zn Mn_2_O_3_	10.19	0.00375
10%Zn Mn_2_O_3_	9.21	0.00575

The estimated micro strain values are listed in Table 2 where micro strain increases with higher Zn content. The doping with Zn creates a ZnMn₂O₄ structure where the oxidation states are Zn²⁺ and Mn³^+^. forming coordination geometry (Tetrahedral vs. Octahedral) where Zn²⁺ prefers tetrahedral coordination (ZnO₄ units) while Mn³⁺ prefers octahedral coordination (MnO₆ units). Therefore, the ionic radius of Zn²⁺ (tetrahedral) is larger compared to Mn³⁺ (octahedral) with ionic radius. Ionic radii differences are significant enough to introduce micro strains and lattice distortions with Zn doping. These effects can lead to observable changes in lattice parameters (reducing particle size). As Zn doping increases, many substitutions occur and the cumulative effect leads to an overall microstrain in the lattice which in turn reduces the lattice parameters (reduces particle size)^35,36^. Thus, the crystallite size of Zn-doped manganese oxide samples decreases with increasing Zn concentration^15^.

### FTIR analysis

**Fig. 2:**
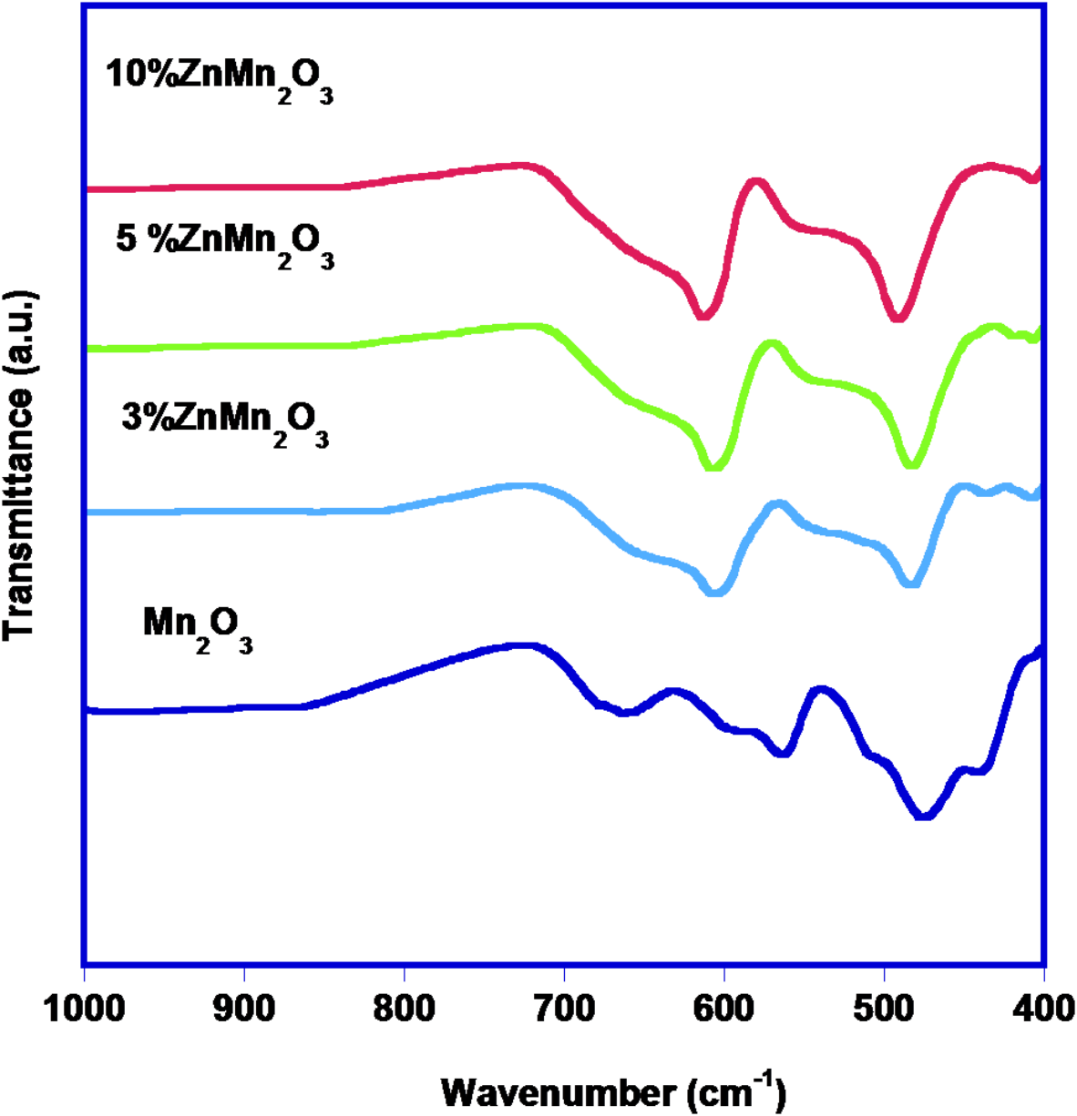
FTIR spectra of pure manganese oxide NPs and doped samples with Zn (3%, 5%, and 10%).

IR spectra of all prepared samples are illustrated in Fig. 2, where the spectral bands of pure Mn_2_O_3,_ which appeared at 700 –400 cm^[-1^ are assigned stretching modes of tetrahedral and octahedral sites in Mn–O bonds^37^. For the doped sample with Zn, the same bands appeared with shifting towards a higher wavenumber^38^.

### SEM analysis

**Fig. 3:**
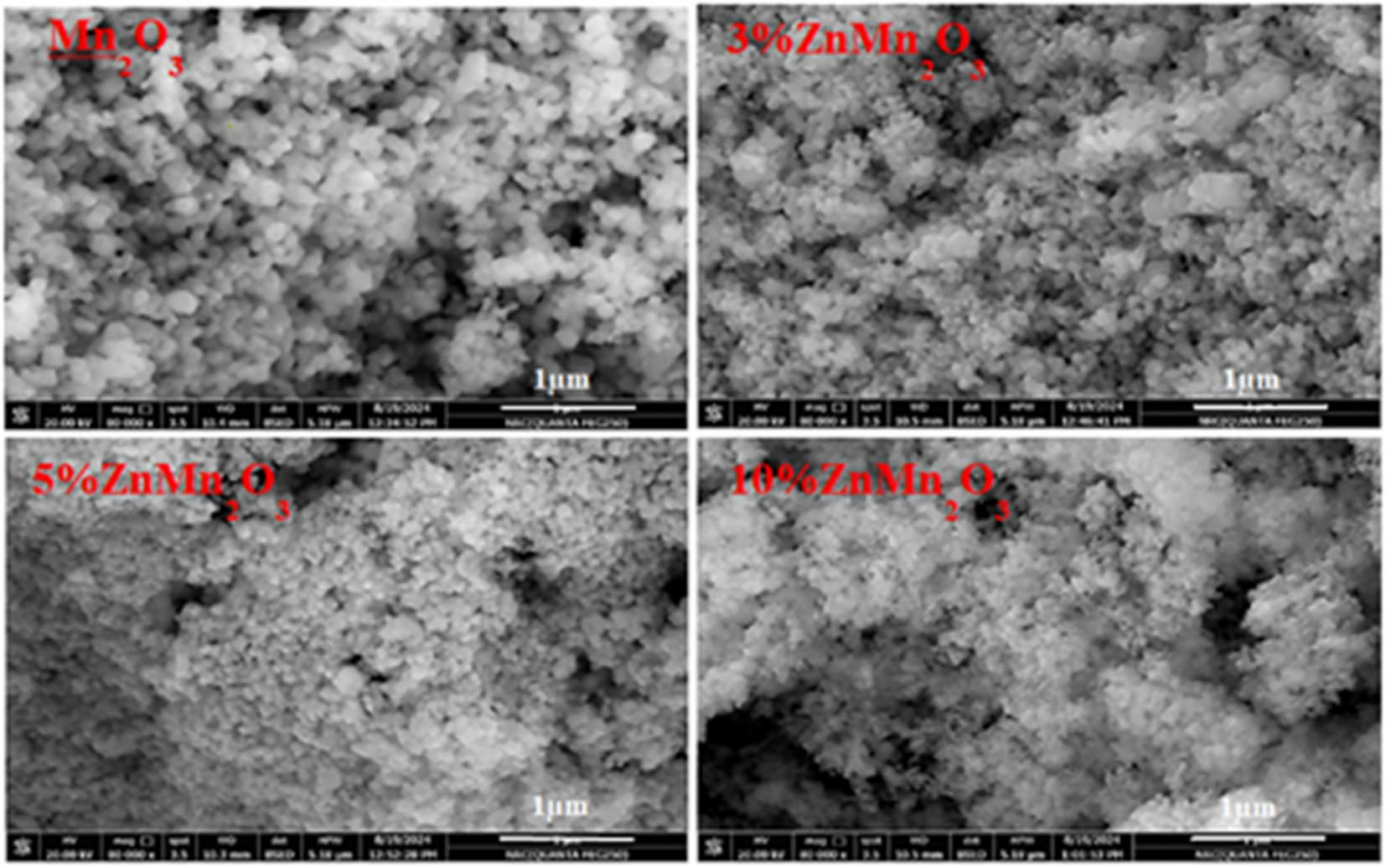
SEM images of pure manganese oxide NPs and doped samples with Zn (3%, 5%, and 10%).

Figure 3 shows the FESEM images of pure Mn_2_O_3_ NPs and Zn-doped samples. The pure sample shows round and cubic-shaped NPs. The FESEM images for the doped samples with Zn (3, 5, and 10%) reveal the transformation of the cubic phase to the tetragonal phase. So as Zn doping increases from 5 to 10%, the grain size declines, and the accumulation rises due to nanoparticles nature with the formation of a spongy structure with very fine pores represented as dark spots in the (10% ZnMn_2_O_3_) sample. Such structure was formed due to the difference between the atomic radius of Zn and Mn ions, as previously discussed in the x-ray. Therefore, the doping level of Zn highly affects the morphology and grain size of the synthesized NPs. The observed change in the structure of the doped samples was previously reported^39–42^.

### Elemental and compositional analysis

**Fig. 4:**
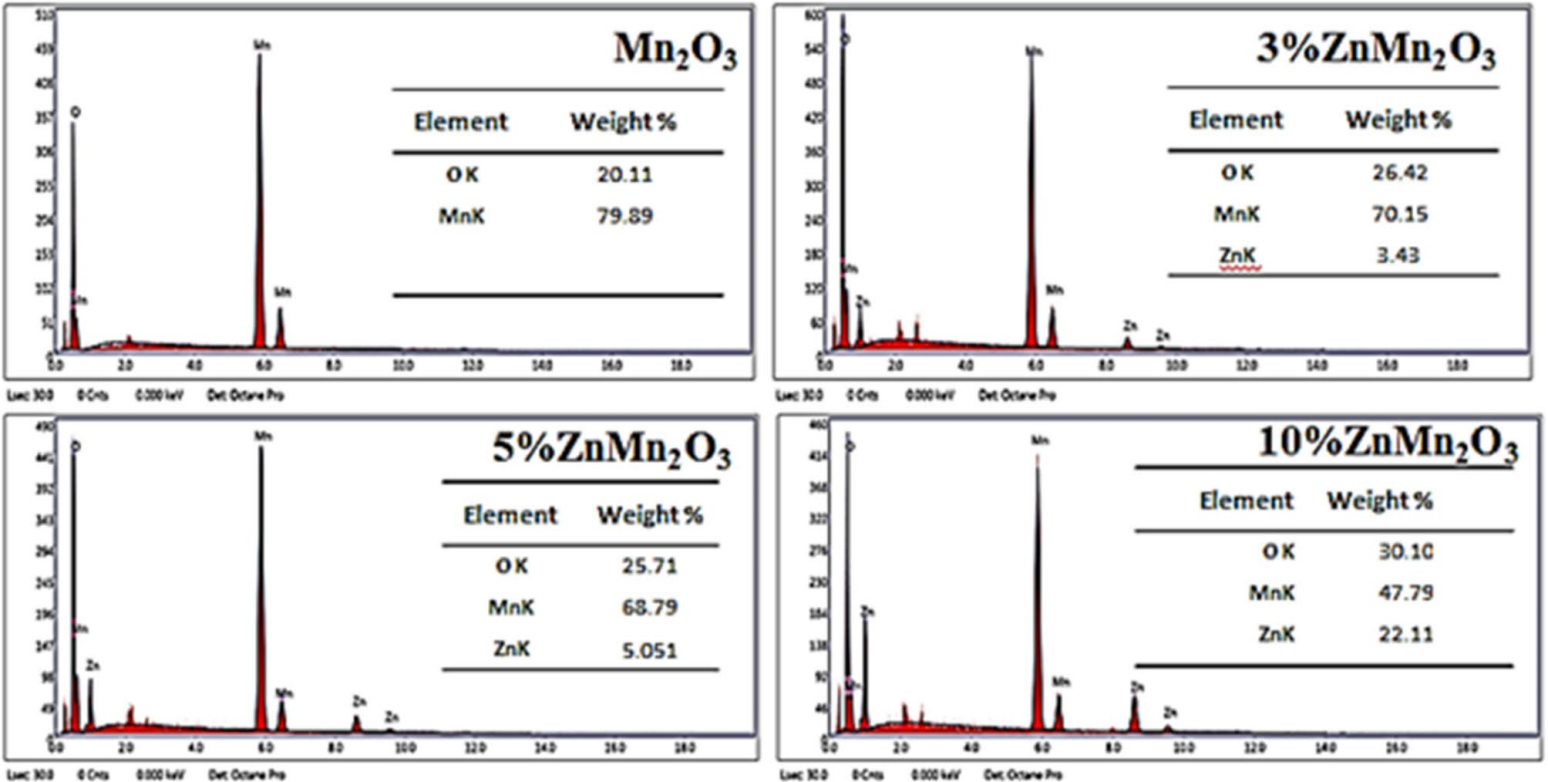
EDX spectrum of pure manganese oxide NPs and doped samples with Zn (3%, 5%, and 10%).

Figure 4 depicts the energy dispersive x-ray (EDX) analysis of pure Mn_2_O_3_ NPs and doped samples with Zn. It is seen that the amount of Zn element rises in the synthesized nanoparticles according to increasing Zn content, confirming the incorporation of Zn in the Mn_2_O_3_ structure. The appearance of low-energy peaks in the range of 10–30 eV can originate from plasmon losses, which occur due to collective oscillations of conduction electrons when X-rays interact with the material. The elemental analyses of Mn, Zn, and O for the investigated samples are clearly shown in the inset of figures for each sample.

### TEM analysis

**Fig. 5:**
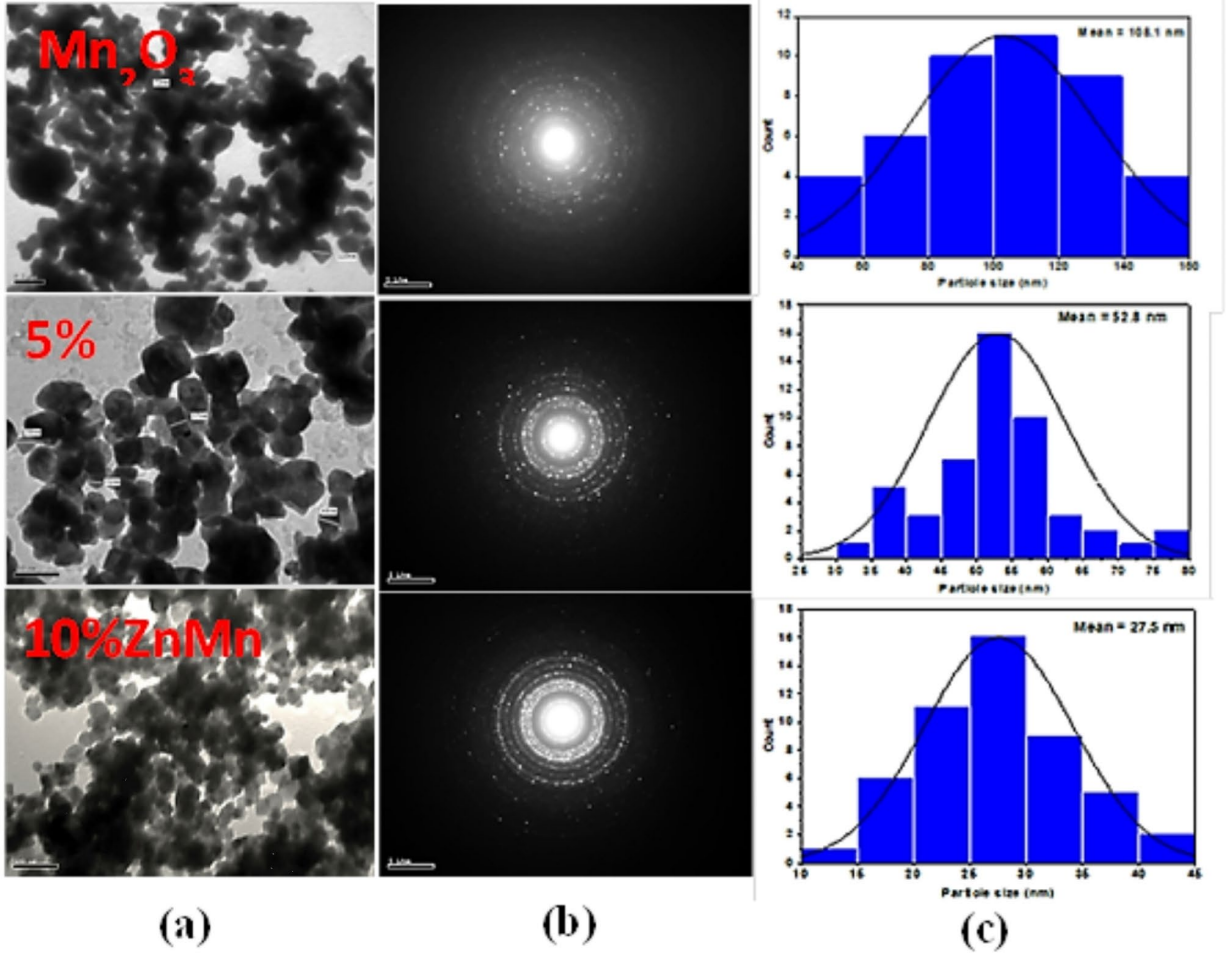
(**a**) TEM images of pure manganese oxide NPs and doped samples, (**b**) their selected area electron diffraction patterns (SAED) and (**c**) corresponding histograms respectively.

TEM photographs of the synthesized nanoparticles are exhibited in Fig. 5**(a**). It reveals the formation of well-defined cubic Mn_2_O_3_ crystals in the un-doped sample, and the particle size varies in the 42–110 nm range. The substitution with Zn has a substantial impact on the size and morphology of the prepared NPs where the doping with Zn leads to the change of morphology of crystal structure from cubic phase to tetragonal phase as observed for doped samples 5% Zn Mn_2_O_3_ and 10% Zn Mn_2_O_3_ with particle size in the range 30–42 nm and from 5 to 30 nm, respectively. The contraction in the particle size of doped samples may be due to the difference between the atomic radius of Zn and Mn ions, as previously discussed in the x-ray. By examining more than 50 particles in captured TEM micrographs using an image analyzer (Image J, 1.42 q created by NIH Bethesda, Maryland, USA), the size distribution of nanoparticles [Fig. 5**(c)**] was determined.

The selected area electron diffraction (SAED) reveals rings patterns with very fine spots for un-doped and doped samples respectively. The ring patterns showed polycrystalline structures for all investigated samples with very fine nanoparticles in addition to the improvement of crystallinity with doping as discussed previously from x-ray analysis **[**Fig. 5**(b)].**

### Optical measurements

**Fig. 6:**
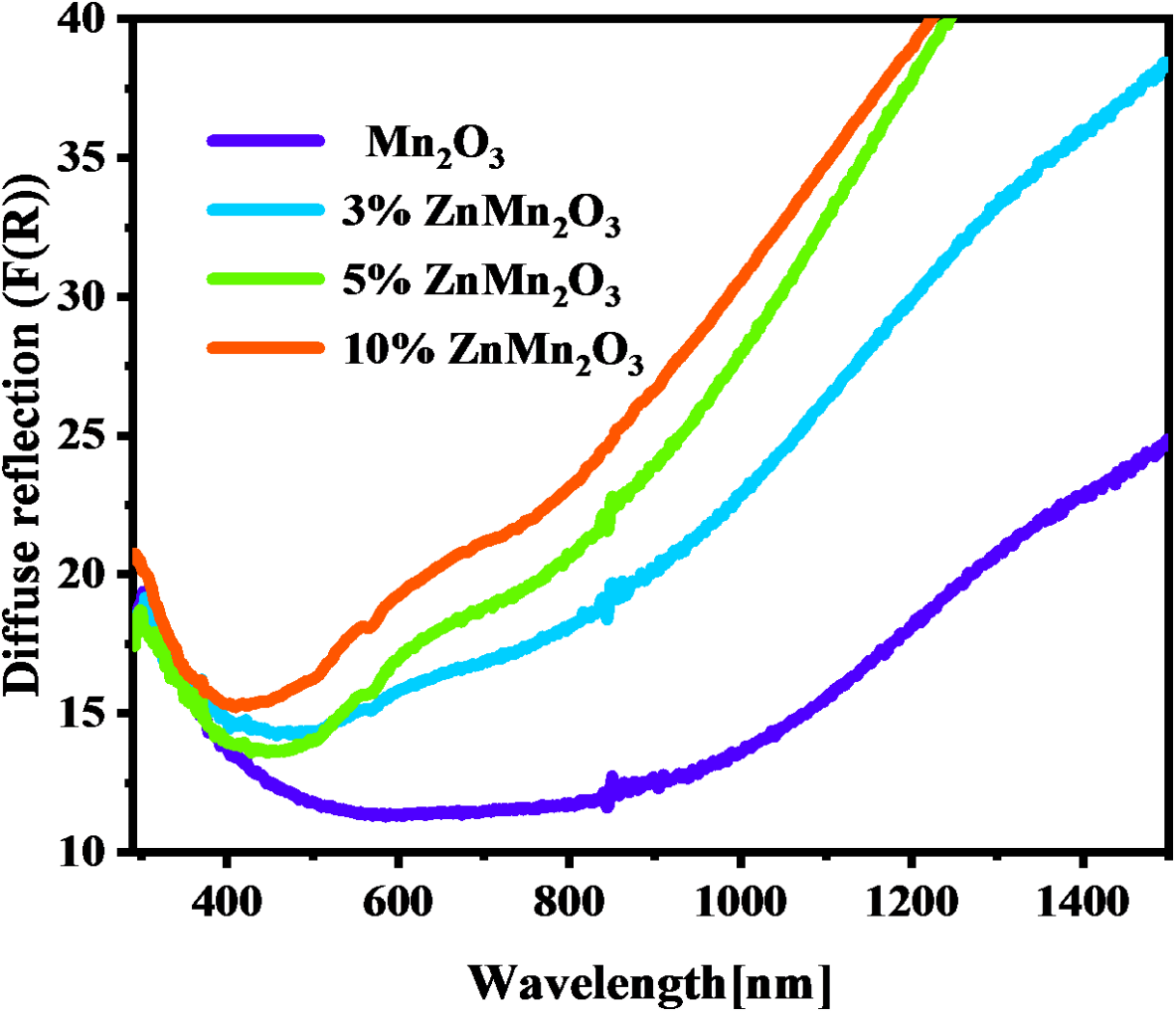
The diffuse reflection F(R), dependence on the wavelength of Mn_2_O_3_ NPs and doped samples with Zn (3%, 5%, and 10%).

The optical properties of synthesized nanoparticles Mn_2_O_3_ and doped samples with Z were studied using diffuse-reflectance measurements. Figure 6 illustrates UV-visible diffuse reflection spectra of Mn_2_O_3_ NPs and doped samples with Zn (3%, 5%, and 10) in the UV - Vis range [200–800 nm] and near IR (NIR). There is a clear dependence of diffuse reflectance on the wavelength with increasing Zn content from 0 to 10%. Also, the results reveal a decrease in the diffuse reflectance between 300 and 500 nm for all investigated samples. This can be interpreted as creating an intermediate state in the energy band gap due to Zn photoluminescence doping^43^.

The optical band gap was evaluated with the diffuse reflectance using Kubelka–Munk function F(R) in Eq. 3 and using Tauc relation as stated in Eq. 4

3 $$\:F\left(R\right)=\frac{{\left(1-R\right)}^{2}}{2R}$$

4 $$\left[ {F\left( R \right)h\upsilon } \right]^{2} = ((1 - R^{2} ))/\left( {2R} \right)^{2} = A(h\upsilon - E_{g} )^{n}$$

Where *R* is the diffuse reflectance, *F(R)* is the Kubelka function used to analyze the absorption spectrum, $\:{E}_{g\:}$is the energy band gap, and n is an integer number use 2 for the direct transition gap and ½ for indirect transition, respectively^44–46^.

**Fig. 7:**
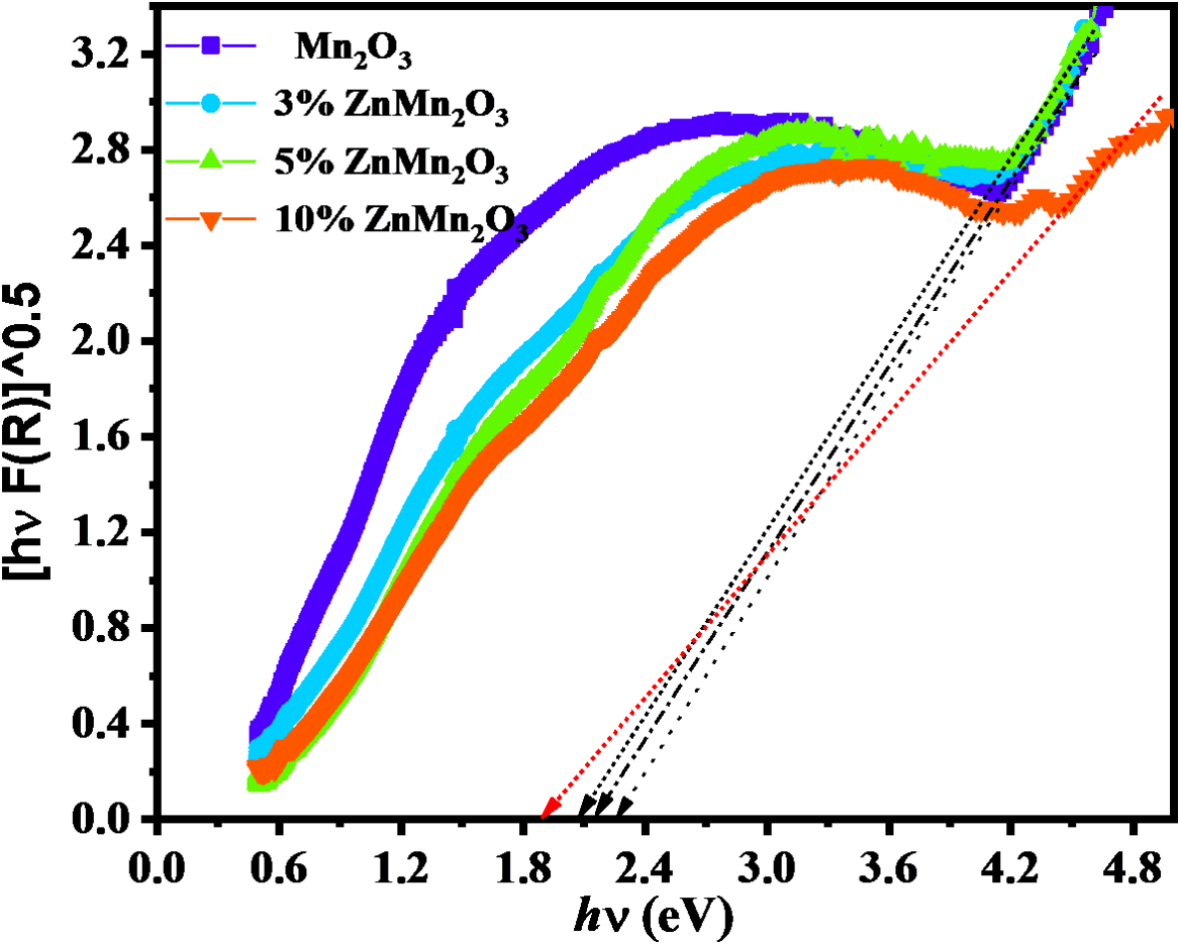
The diffuse reflection F(R), dependence on the wavelength of Mn_2_O_3_ NPs and doped samples with Zn (3%, 5%, and 10%).

The band gap was determined from the intercept on x- the axis (energy) taking the linear part of the plot. Their values were estimated using Eq. 4 as shown in Fig. 7.

It can be observed that all investigated samples show sharp absorption edges in the above-defined wavelength range, and the resulting curves are suggested to be due to indirect interband transitions in the visible region. The extracted band energy gap values are listed in Table 3.

**Table 3:** The energy band gap of Mn_2_O_3_ NPs and doped samples with Zn (3%, 5%, and 10%).

Sample	Energy band gap E_g_ (eV)
Pure Mn_2_O_3_	2.26
3%Zn Mn_2_O_3_	2.15
5%Zn Mn_2_O_3_	2.07
10%Zn Mn_2_O_3_	1.89

The band gap of the prepared samples was manipulated to lower values with increasing Zn doping, and they ranged in the visible wavelength region as previously reported for Mn_2_O_3_. Also, the detected values of energy gaps are due to indirectly allowed transitions that occur via intra-band transitions or indirect transitions that occur within the same energy. The observed red shift in the band gap can be discussed as; with increasing of Zn content, the Zn atoms introduce impurity states in the band structure and create localized states near the band edges which in turn effectively in reducing the energy difference between the conduction band minimum and valence band maximum, leading to band gap narrowing. The degree of band gap narrowing depends on the host material, doping concentration, and defect formation. At very high Zn concentrations, excessive defects or disorder can further modify the electronic structure^47,48^.

**Fig. 8:**
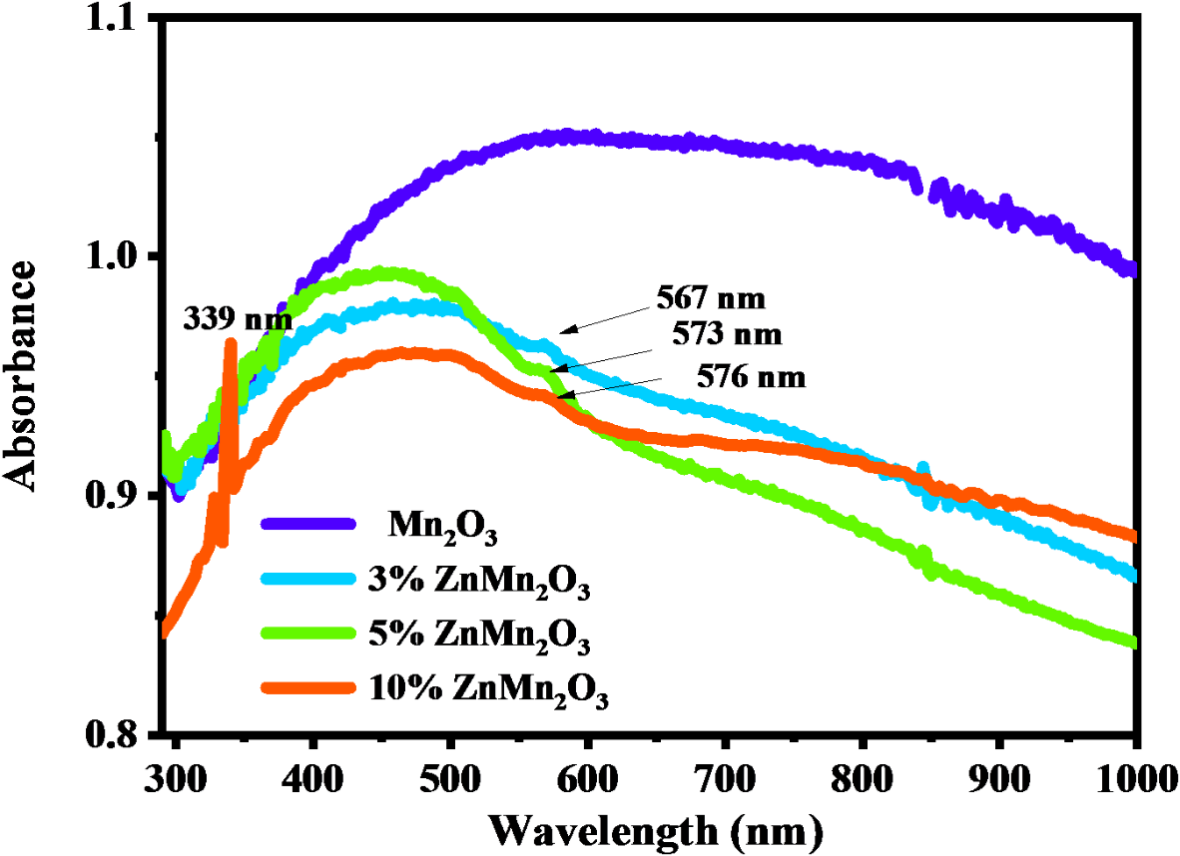
UV–VIS absorption gaps of the synthesized nanoparticles of Mn_2_O_3_ NPs and doped samples with Zn (3%, 5%, and 10%).

The absorbance spectra for Mn_2_O_3_ doped samples with Zn (3, 5, and 10%) are depicted in Fig. 8. The figure reveals strong and sharp absorbance for 10%Zn Mn_2_O_3_ sample in UV region wavelengths at 329 nm and 339 nm due to the absorption band of Zn^49^. Broad absorbance peaks were found for all samples in the Vis region wavelengths. In addition to the appearance of three weak peaks at 567, 573, and 576 nm in all doped samples. With increasing Zn content, the observed band gap energy shifted towards the narrow band energy in the visible region 2.18, 2.16, and 2.15 eV, confirming that the prepared samples can be appropriate as photocatalysts in the visible region^50^. Depending on optical results, 339 nm was taken as an excitation wavelength for photoluminescence measurements.

### Photoluminescence measurements

**Fig. 9:**
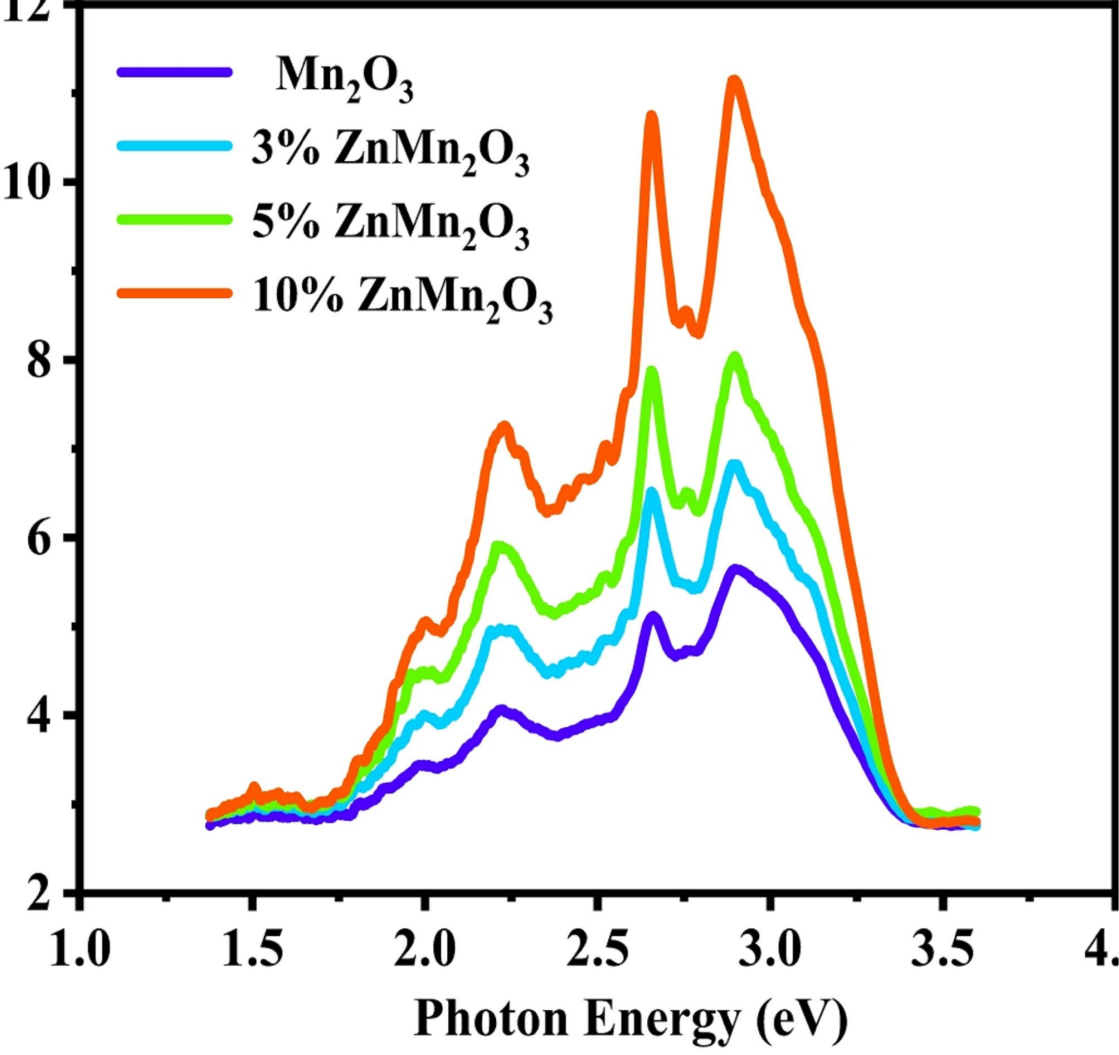
PL spectra of Mn_2_O_3_ and doped samples with Zn (3, 5, and 10%) at excitation wavelength 339 nm at room temperature.

The photoluminescence (PL) approach has been widely utilized to study the structure and characteristics of the active sites on the surface of metal oxides^51,52^ because of its extraordinary sensitivity and non-destructive nature. Additionally, the photocatalysis over the semiconductors field has benefited from applying the PL approach to comprehend surface dynamics. The photochemical and optical properties of semiconductor materials, including surface oxygen vacancies and defects and the effectiveness of charge carrier trapping, migration, and transfer, can all be studied using the photoluminescence (PL) spectrum^53–56^.Therefore, the solid theoretical basis for creating novel, highly active semiconductor photocatalysts and promptly assessing the photocatalytic activity of semiconductor samples can be obtained from the PL spectrum. In actuality, the characteristics of the PL signal mostly dictate the correlations between the PL spectrum and photocatalytic activity. The PL spectrum of pure Mn_2_O_3_ NPs and doped samples with Zn (3, 5, and 10%) at room temperature are revealed in Fig. 9. The spectrum of all investigated samples exhibited four characteristic emission peaks in the visible energy region at 425, 466, 563 and 623 nm with energies 1.99, 2.19, 2.66 and 2.91 eV, respectively are related to Mn_2_O_3_. These peaks are associated with electronic transitions influenced by defect states (such as oxygen vacancies) and the Mn oxidation states within the crystal lattice. The peaks at 425 nm and 466 nm correspond to transitions from shallow donor level such as oxygen vacancies to the valence band that act as electron traps, reducing recombination and enhancing charge separation. While the emission peak at 563 nm is often caused from deep level defects including transitions of Mn^3+^ ions. The intra 3d transitions of Mn^3+^ions in an octahedral crystal field produce such emission. These deep defects generate the reactive oxygen species and efficient charge separation. While, the peak at 623 nm is attributed to the charge transfer between Mn ions and deep level states formed by interstitial Mn ions. These deep level traps prolong carrier lifetimes enhancing the redox reaction which are critical for photocatalytic performance. Additionally, the intensity of visible PL peaks increases with increasing Zn content from 0 to 10% due to increasing the oxygen vacancies and defects that reside on the surface results from the transformation of the Mn_2_O_3_ with cubic structure to ZnMn_2_O_4_ with tetragonal structure after Zn doping as previously discussed from the XRD investigation. Moreover, the peak positions of PL signals remain relatively constant. However, this suggests several relatively stable exciton energy levels, oxygen vacancies, defects, and surface states^57^.

A common and effective modification is doping with metal ion doping, which further enhances semiconductor materials’ photocatalytic performance. Doping with Zn^2+^ ions, which in its stable chemical state has a semi-filled or wholly filled outer shell in electronic structure, like structure cannot capture electrons, despite, it can significantly affect the surface structure such as defects and oxygen vacancies and consequently change the PL spectra.

The visible photoluminescence emissions PL revealed in the band energy range [1.5–3 eV] are due to free excitons emission and the transition in defect states due to oxygen vacancy^58,59^.

It was previously reported that PL emissions of Zn NPs revealed near-band-edge and visible luminescence due to the exciton transitions where the excitonic PL signal increases with Zn doping. As a result, the efficiency of the recombination process decreases due to the emission of photons when an exciton recombines. Moreover, in such an indirect band gap structure, the recombination process is less efficient due to the deficiency of phonons, where the presence of phonons is the most responsible factor for efficient recombination^60^. Depending on the PL obtained results, the doping with Zn improved the photocatalytic activity of Mn_2_O_3_.

### Photocatalytic activity

**Fig. 10:**
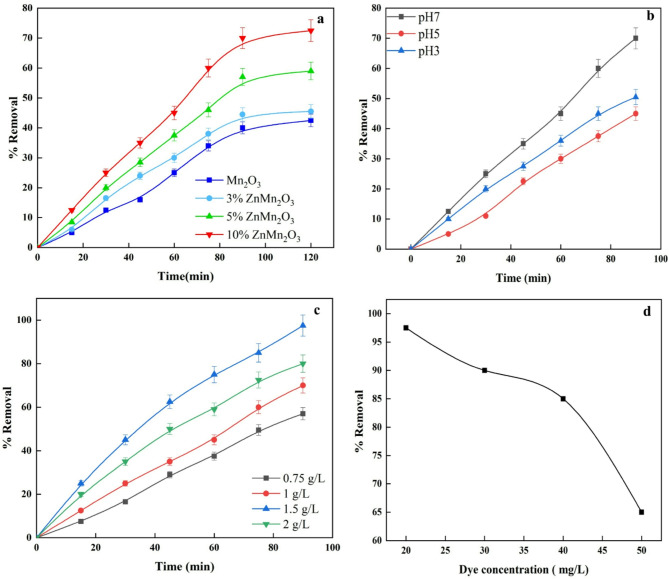
(**a**) The irradiation time impact, (**b**) The pH impact, (**c**) The dose impact of prepared catalysts, (**d**) The initial dye concentration impact on the photocatalytic efficiency for 10% ZnMn_2_O_3_.

The efficiency of pure Mn_2_O_3_ and doped samples with Zn in MG degradation is assessed by comparing the concentration drop to the initial concentration level. Figure 10a shows the degradation percentage of MG as a function of the exposure period of the investigated photocatalysts to visible light. The results showed that MG deterioration started quickly after interacting with samples and worsened over time till become nearly constant which consider maximum value^61^. The dye breakdown could be due to the chromophores responsible for its color. After 90 min at pH 7 and a catalyst weight of 1 g/L, the degradation percentages with Mn_2_O_3_, 3% ZnMn_2_O_3_, 5% ZnMn_2_O_3_, and 10% ZnMn_2_O_3_ were 40%, 44.5%, 57%, and 70%, respectively. At 120 min, there was a slight rise in the degradation percentage. We settled on a 90-minute light-illumination routine^62^. Very little dye is also lost (1% in an illuminated blank solution without a catalyst). Also, in a control degradation experiment, doped samples with Zn could remove about 3% of the dye without light.

The degradation results are matching with the values of band gap energies of Mn_2_O_3_, 3% ZnMn_2_O_3_, 5% ZnMn_2_O_3_, and 10% ZnMn_2_O_3_ that moved towards the narrow band energy at 2.26, 2.15, 2.07, and 1.89 eV, respectively (Table 3) and increasing the oxygen vacancies and defects that reside on the surface results from the transformation of the Mn_2_O_3_ with cubic structure to ZnMn_2_O_4_ as confirmed in the intensity of visible PL peaks increases with increasing Zn content from 0 to 10%.

As most the metal oxide the mechanism of pollutant degradation depends on, the ease with which electrons emitted by visible light migrate from the valence band to the conduction band causes photodegradation. O_2_ molecules generate oxygen radicals ($\:{O}_{2}^{.-}$) to scavenge electrons in the conduction band. In addition, valence band holes oxidize hydroxyl ions and H_2_O, creating$\:\:{\text{O}\text{H}}^{.}$ responsable for degrade the pollutant^63^.The Eqs. (5–9) simplify radical production and degradation.

5 $${\text{ZnM}}{{\text{n}}_{\text{2}}}{{\text{O}}_{\text{3}}}+{\text{h}}\upsilon (\uplambda >{\text{4}}00{\text{ nm}}) \to {\text{ZnM}}{{\text{n}}_{\text{2}}}{{\text{O}}_{\text{3}}}\left( {{{\text{e}}_{{\text{cb}} - }}+{{\text{h}}_{{\text{vb}}}}} \right)$$

6 $${\text{ZnM}}{{\text{n}}_{\text{2}}}{{\text{O}}_{\text{3}}}\left( {{{\text{e}}_{{\text{cb}} - }}} \right)+{{\text{O}}_{\text{2}}} \to {\text{ZnM}}{{\text{n}}_{\text{2}}}{{\text{O}}_{\text{3}}}+O_{2}^{{ \cdot - }}$$

7 $${{\text{h}}^+}+{{\text{H}}_{\text{2}}}{\text{O}} \to {\text{O}}{{\text{H}}^ \cdot }+{\text{H}}$$

8 $${{\text{e}}^ - }+{{\text{O}}_{\text{2}}} \to O_{2}^{{ \cdot - }}$$

9 $${{\text{h}}_{{\text{vb}}+}}+{\text{dye}} \to {\text{degradation by products}}$$

The impact of pH on the catalytic activity toward the MG dye is illustrated in Fig. 10b. Using H_2_SO_4_ and NaOH allowed the dye solution’s pH to be varied between 3 and 7. Both the high acidic and basic pH levels showed low rates of photodegradation response. The photodegradation rate was most significant when the pH was 7. The experiment of pH 8 is not considered since Methyl green precipitated at a pH of 8^64,65^.Based on this result, it was found that pH = 7 is ideal. This suggests that hydroxyl radical generation is significantly boosted in neutral conditions, which in turn helps to increase the reaction rate. Conversely, the dye removal reduce in highly acidic media since H ions give metal oxide a positive charge and protonate cationic dyes at low pH^63^. Dye molecule repulsion from the semiconductor reduces photocatalytic degradation. Photocatalytic degradation increases as pH rises because dye-semiconductor repulsion reduces. For 10% ZnMn_2_O_3_, the MG % removal in the acidic medium was 50.5%, while for pH 7, it was 70%.

Figure 10c demonstrates that the percentage of MG removal is affected by the dose of photocatalyst. At pH 7 and irradiation time 90 min, it was found that photodegradation increases as the dose of 10% ZnMn_2_O_3_ increases from 0.75 to 2 g/L. The photodegradation efficacy of the MG dye is increased from 57 to 97.5% as result of, As the loading concentration of a photocatalyst rises, more active sites are generated on its surface, making it easier to generate hydroxyl radicals when exposed to light^39^. At 1.5 g/L, the dye degradation performance peaked, and this dosage is thought to be ideal for photocatalytic degradation^66^. Nevertheless, when the concentration was 2 g/L, the elimination percentage fell to 80%. This is because the dark solution prevented light from reaching the surface active sites, which inhibited photocatalytic degradation^61^. as well, the collision rate of particles rises with density. Blocking light absorption reduces the metal oxide’s capacity to release hydroxyl radicals^63^.

Figure 10d displays the photocatalytic effectiveness of 10% ZnMn_2_O_3_ in degrading MG dye at pH 7, starting concentrations ranging from 20 to 50 mg/L, and a catalyst dose of 1.5 g/L. This was seen after 90 min of visible light irradiation. At a concentration of 20 mg/L MG, 10% ZnMn_2_O_3_ photodegraded 97.5% of the dye, and at 30 mg/L, 90% of the dye. Nonetheless, the photocatalytic activity dropped to 85% and 65%, respectively, when the initial concentration of MG was increased to 40 and 50 mg/L. The ideal dose of dye for future degradation investigations is 20 mg/L, which is also the MG degradation efficiency peak. More efficiency may be attributable to the fact that, at lower dye concentrations, most of the active sites on the catalyst surface are empty. However, degradation efficiency drops when the initial dye concentration is high because the catalyst’s active sites are nearly filled with dye molecules, and light photon penetration drops^61^. Lead to A decline hydroxyl radical number, which means fewer attacks on dye molecules and slower decomposition.

The experimental data has been fitted into the pseudo-first-order kinetic model Langmuir-Hinshelwood to estimate the kinetics of MG degradation utilizing all prepared samples^67,68^.

**Fig. 11:**
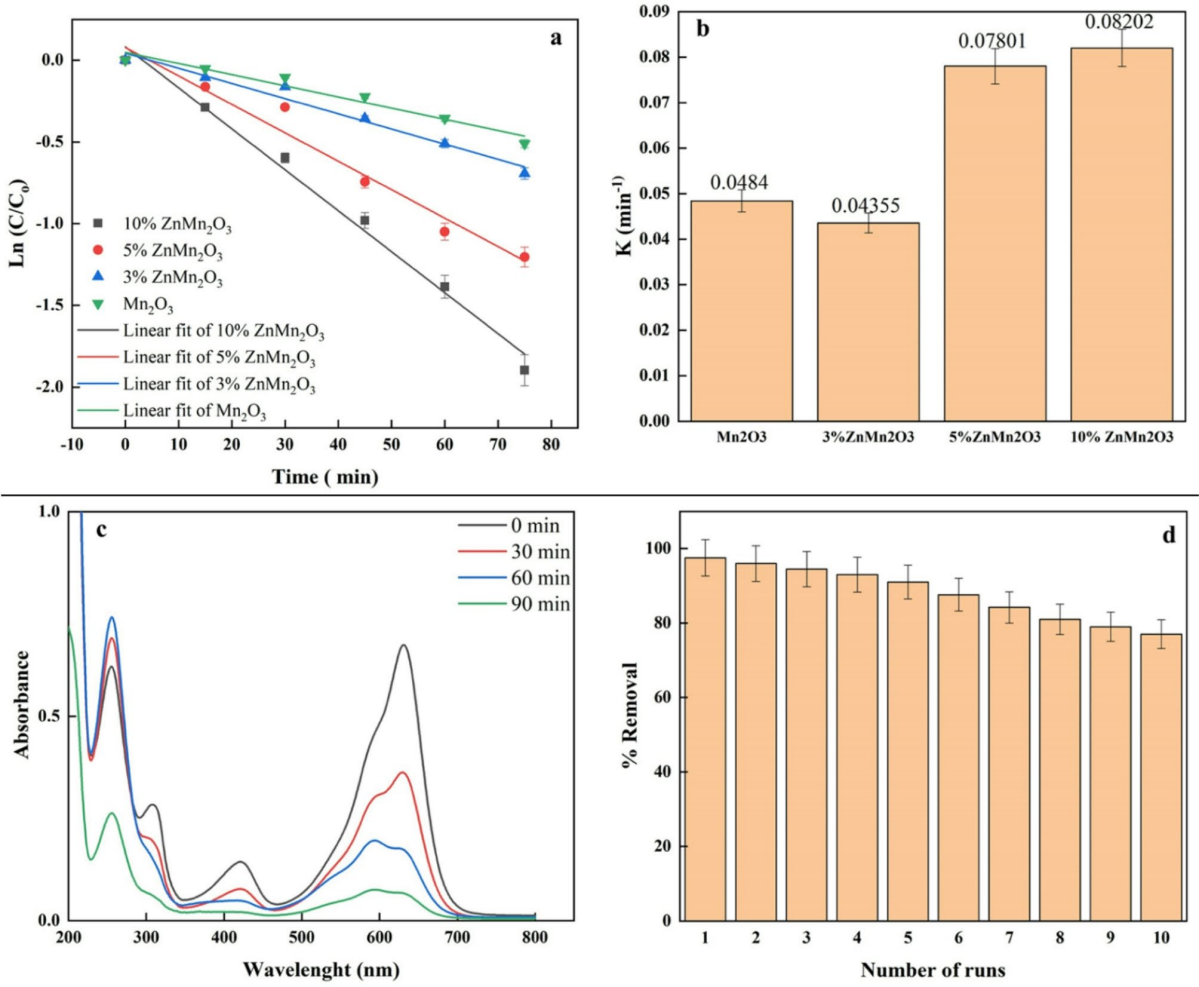
(**a**) Pseudo-first-order kinetics study for the photocatalytic efficiency, (**b**) Rate constant of Pseudo first-order kinetics, (**c**) Time-dependent UV–Vis absorption spectra of the photocatalytic degradation (**d**) Recycle efficiency of 10% ZnMn_2_O_3_ for MG degradation dye.

Figure 11a shows the results of a linear regression plotting ln(C/C_0_) against the irradiation period (in minutes), which indicates that the pseudo-first-order kinetic model is a good fit^39^. Kinetic rate constants (k) for photodegradation of MG dye are also displayed in Fig. 11b. The lowest band gap for 10% ZnMn_2_O_3_ was corroborated by the values of the rate constants, which indicate that the maximum rate constant for the degradation of MG dye is 0.08202 min^[-1^.

The absorption spectra of methyl green dye at various time intervals in the presence of 10% ZnMn_2_O_3_ as a photocatalyst are displayed in Fig. 11c. The results indicate a prominent peak at 626 nm for a 20 mg/L methyl green dye solution in water exposure to visible light at a photocatalyst dose of 1.5 g/L, and the intensity of these peaks gradually decreases as exposure time increases. This proves that methyl green degrades when a 10% ZnMn_2_O_3_ photocatalyst is present^69^.

For photocatalysts to have any real-world application, they must be stable and reusable, two of the most critical sustainability metrics. We have completed 10 MG decolorization cycles over 10% ZnMn_2_O_3_ with our optimized working conditions. The percentage of dye (MG) removed was 97.5% removed on the first and just 77% removed on the run number 10. The recycling test showed a little drop in removal effectiveness (Fig. 11d), which could have been caused by the catalyst being lost slightly during washing and filtration.

Table 4 compares the most active sample 10% ZnMn_2_O_3_ to numerous catalysts’ MG dye degradation performance in previous investigations. This study surpasses previous studies using Ag^+^ Doped ZnO, CuONPs, nitrogen-doped carbon quantum dots (NCQDs), and CuFe_2_O_4_ doped Ti to degrade Methyl green by various % removal depending on initial dye concentration and light source. 10% ZnMn_2_O_3_ effectiveness makes it a promising environmental remediation material, notably for organic dye degradation with respect to using higher initial dye concentration under visible radiation, which is cheaper and more sustainable than UV light.

**Table 4:** Assessment of MG degradation with 10% ZnMn_2_O_3_ in comparison to previous research.

Catalyst	Removal (%)	Light type	Time (min)	Dye concentration (mg/L)	References
Ag doped ZnO	85.9	UV-Vis	30	14	^70^
CuO NPs	65	Sun light	60	10	^34^
Nitrogen-doped carbon quantum dots	99	UV	20	10	^71^
Ti doped CuFe_2_O_4_	92	LED	60	10	^72^
10% ZnMn_2_O_3_	97.5	Visible	90	20	Current work

### Antibacterial activity

**Fig. 12:**
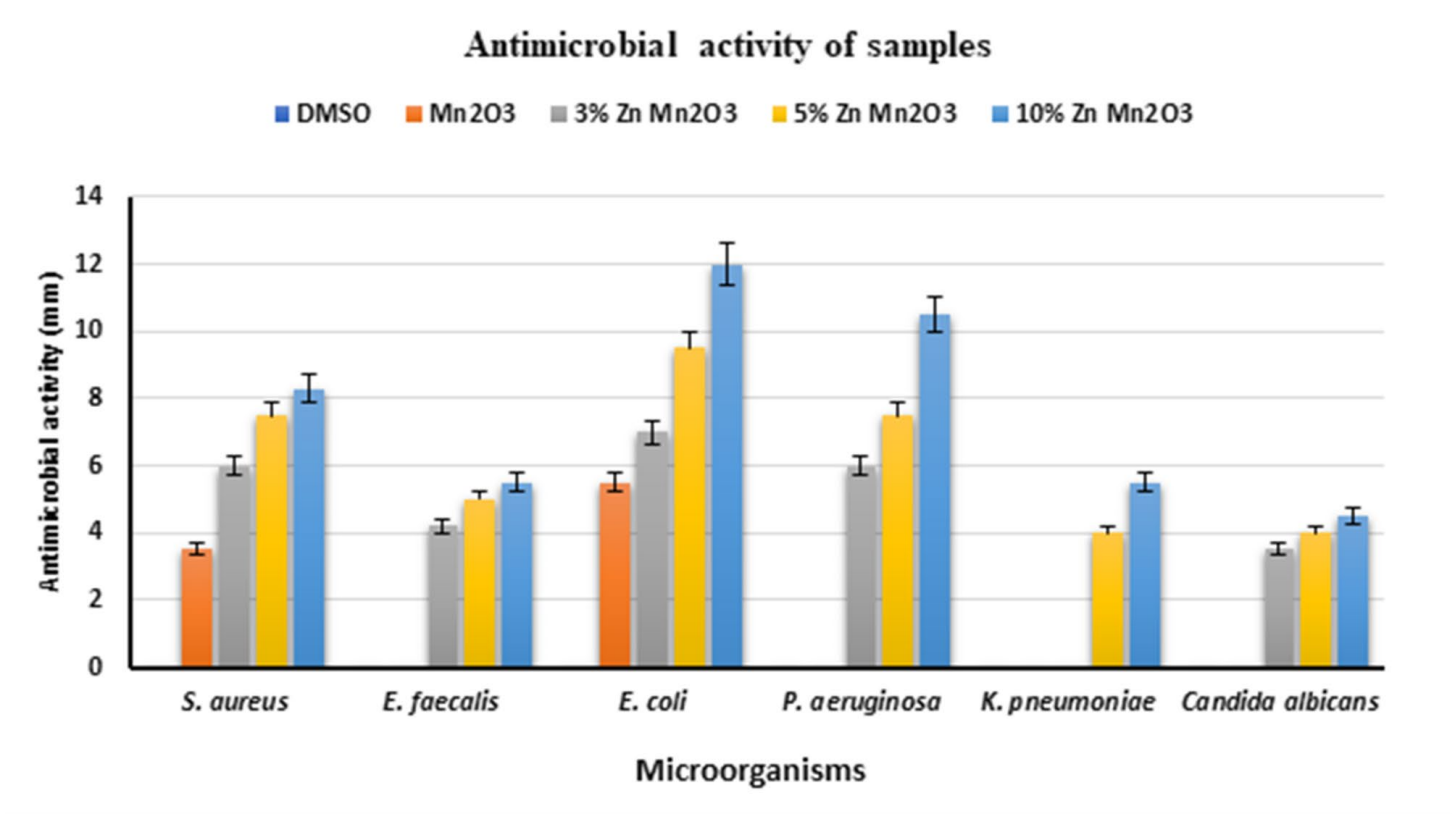
Antimicrobial activity of samples evaluated by well diffusion method against *S. aureus* ATCC25923, *E. faecalis* ATCC29212, *K. pneumoniae* ATCC25175, *P. aeruginosa* ATCC10145, *E. coli* ATCC25915 and *Candida albicans.*

**Fig. 13:**
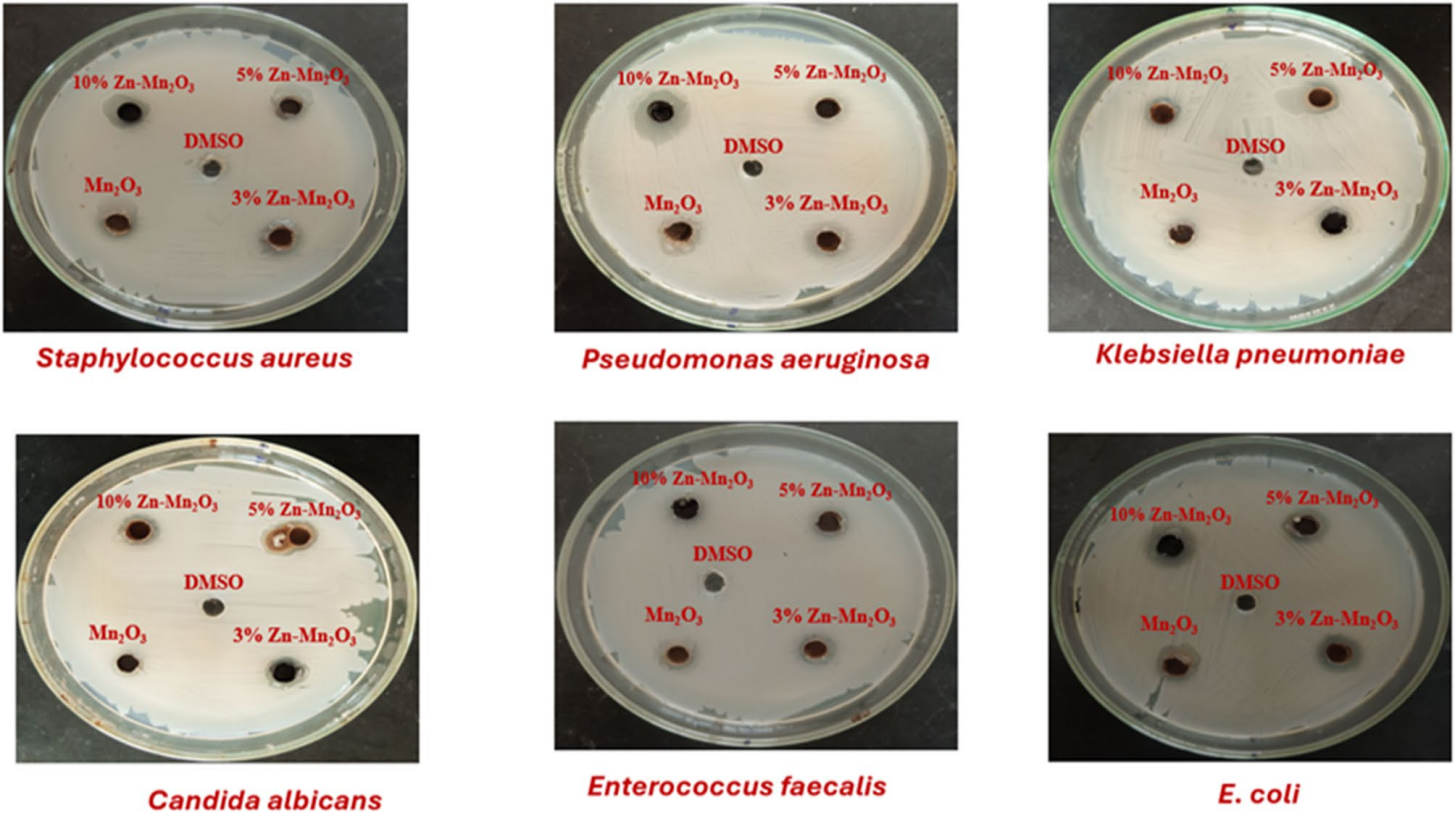
Antimicrobial activity of samples evaluated by well diffusion method against *S. aureus* ATCC25923, *E. faecalis* ATCC29212, *K. pneumoniae* ATCC25175, *P. aeruginosa* ATCC10145, *E. coli* ATCC25915 and *Candida albicans*.

The antimicrobial properties of the various samples containing different concentrations of zinc-doped manganese oxide (pure Mn_2_O_3_, 3% Zn Mn_2_O_3_, 5% Zn Mn_2_O_3,_ and10% Zn Mn_2_O_3_) were evaluated against both gram-negative bacteria (*E. coli*,* Klebsiella pneumoniae*, and *P. aeruginosa*) and gram-positive bacteria (*S. aureus* and *Enterococcus faecalis*). The investigation revealed that the samples containing 3%, 5%, and 10% zinc-doped manganese oxide exhibited the most pronounced antibacterial effects, with measurements of 6.00 ± 0.10, 7.50 ± 0.10, and 10.50 ± 0.10 against *P. aeruginosa* ATCC10145 and 7.00 ± 0.10, 9.50 ± 0.0, and 12.00 ± 0.08 against *E. coli* ATCC25915, as illustrated in Fig. 12; Table 5. Moreover, our findings indicated that these samples (3% Zn Mn_2_O_3_, 5% Zn Mn_2_O_3_ and 10% Zn Mn_2_O_3_) demonstrated comparable antibacterial activity, registering 6.00 ± 0.10, 7.50 ± 0.10, and 8.30 ± 0.05 against *S. aureus* ATCC25923 and 4.25 ± 0.0, 5.00 ± 0.05, and 5.50 ± 0.01 against *E. faecalis* ATCC29212. Specifically, the samples containing 5% and 10% zinc-doped Mn_2_O_3_ displayed significant inhibition, with zone of inhibition measurements of 7.50 ± 0.10 and 8.30 ± 0.05 mm for *S. aureus* ATCC25923, 5.00 ± 0.05 and 5.50 ± 0.01 mm for *E. faecalis* ATCC29212, 4.00 ± 0.10 and 5.50 ± 0.05 mm for *K. pneumoniae* ATCC 25,175, 7.50 ± 0.10 and 10.50 ± 0.10 mm for *P. aeruginosa* ATCC10145, 9.50 ± 0.0 and 12.00 ± 0.08 mm for *E. coli* ATCC25915, and 4.00 ± 0.00 and 4.50 ± 0.10 mm for *Candida albicans*. In contrast, the inhibitory effects of the 3% zinc-doped Mn_2_O_3_ samples were moderate, measuring 6.00 ± 0.10 mm for *S. aureus* ATCC25923, 4.25 ± 0.00 mm for *E. faecalis* ATCC29212, 6.00 ± 0.10 mm for *P. Aeruginosa* ATCC10145, 7.00 ± 0.10 mm for *E. coli* ATCC25915, and 3.50 ± 0.10 mm for *Candida albicans*. Notably, only the sample containing pure Mn_2_O_3_ exhibited inhibitory effects against *S. aureus* ATCC25923 and *E. coli* ATCC25915, measuring 3.50 ± 0.20 and 5.50 ± 0.10 mm, respectively. Consequently, our results suggest that the doped samples (5% Zn-Mn_2_O_3_, and 10%Zn-Mn_2_O_3_) demonstrate promising antimicrobial activity against the tested pathogens, as illustrated in Fig. 13. It is worth emphasizing that zinc can effectively combat pathogenic bacteria by disrupting bacterial cell membranes, resulting in the leakage of cellular contents and eventual cell death. Similar studies have also supported the antimicrobial efficacy of ZnO nanomaterials against pathogenic bacteria. On the other hand, the antimicrobial activities of the most active samples (5%Zn-Mn_2_O_3_ and 10%Zn-Mn_2_O_3_) were also tested to determine the minimum inhibitory concentration (MIC), the MIC values of (5%Zn-Mn_2_O_3_ and 10%Zn-Mn_2_O_3_) against pathogenic bacteria were determined and tabulated (Table 6). The results of MIC for (5%Zn-Mn_2_O_3_ and 10%Zn-Mn_2_O_3_) were (10 mg/mL) toward *E. coli* ATCC25915 and *P. aeruginosa* ATCC10145, respectively. So, the antimicrobial activity is influenced by the Zn content doped into the prepared samples. Similar reports were performed by *Darwich et al. and Ekram et al.*, who conducted analogous investigations exploring the antimicrobial properties of material nanoparticles^73,74^. The synergy between photocatalysis and antibacterial activity stems from the role of ROS in oxidizing organic matter and pathogens. By designing materials that maximize ROS generation under light, we create multifunctional solutions for sustainability and healthcare. This dual focus reflects a growing trend in materials science to develop smart systems that tackle interconnected global challenges. Mechanism of Action of Zn-doped Mn₂O₃ Release of Zn²⁺ Ions: Zinc ions disrupt bacterial cell membranes, interfere with enzyme function, and induce oxidative stress. Photocatalytic activity of Mn₂O₃ generates ROS which damage cell components (lipids, proteins, DNA). Synergistic Effect: Zn doping enhances ROS generation and ion release, improving antimicrobial efficacy. Similarly, Sm-doped ZnO-SnO2 NPs enhanced photodegradation and antibacterial properties, driven by improved charge separation, visible light absorption, and ROS generation, make them ideal for tackling pollution and microbial contamination^75^.

**Table 5:** Antimicrobial activity of samples evaluated by well diffusion method.

Microorganisms	Type	Antimicrobial activity (mm)				
		DMSO	Mn_2_O_3_	3% Zn-Mn_2_O_3_	5% Zn-Mn_2_O_3_	10% Zn-Mn_2_O_3_
*S. aureus* ATCC25923	G + ve	(−)	3.50 ± 0.20	6.00 ± 0.10	7.50 ± 0.10	8.30 ± 0.05
*E. faecalis* ATCC29212	G + ve	(−)	(−)	4.25 ± 0.00	5.00 ± 0.05	5.50 ± 0.01
*K. pneumoniae* ATCC 25,175	G −ve	(−)	(−)	(−)	4.00 ± 0.10	5.50 ± 0.05
*P. aeruginosa* ATCC10145	G −ve	(−)	(−)	6.00 ± 0.10	7.50 ± 0.10	10.50 ± 0.10
*E. coli* ATCC25915	G −ve	(−)	5.50 ± 0.10	7.00 ± 0.10	9.50 ± 0.0	12.00 ± 0.08
*Candida albicans*	**–**	(−)	(−)	3.50 ± 0.10	4.00 ± 0.00	4.50 ± 0.10

The biological experiment was carried out in triplicate. Values are given as mean ± standard error.

(G + ve) Gram-Positive, (G − ve) Gram-Negative, (−) Negative.

**Table 6:** MIC of the most active samples by agar diffusion method.

No.	Sample	Conc. mg/mL		Antibacterial activities (mm)				
			E. coli ATCC25915	S. aureus ATCC25923	E. faecalis ATCC29212	*P*. aeruginosa ATCC10145	K.pneumoniae ATCC 25,175	Candida albicans
1		20	(+) 5.50 ± 0.10	(+) 3.50 ± 0.20	(−)	(−)	(−)	(−)
2	Mn_2_O_3_	10	(−)	(−)	(−)	(−)	(−)	(−)
3		5.0	(−)	(−)	(−)	(−)	(−)	(−)
4		20	(+) 7.00 ± 0.10	(+) 6.00 ± 0.10	(+) 4.25 ± 0.00	(+) 6.00 ± 0.10	(−)	(+) 3.50 ± 0.00
5	3% Zn Mn_2_O_3_	10	(−)	(−)	(−)	(−)	(−)	(−)
6		5.0	(−)	(−)	(−)	(−)	(−)	(−)
7		20	(+) 9.50 ± 0.0	(+) 7.50 ± 0.10	(+) 5.00 ± 0.05	(+) 7.50 ± 0.10	(+) 4.00 ± 0.10	(+) 4.00 ± 0.10
8	5% Zn Mn_2_O_3_	10	(+) 4.51 ± 0.0	(−)	(−)	(−)	(−)	(−)
9		5.0	(−)	(−)	(−)	(−)	(−)	(−)
10		20	(+) 12.00 ± 0.08	(+) 8.30 ± 0.05	(+) 5.50 ± 0.01	(+) 10.50 ± 0.10	(+) 5.50 ± 0.05	(+) 4.50 ± 0.10
11	10% Zn Mn_2_O_3_	10	(+) 5.00 ± 0.10	(−)	(−)	(+) 3.00 ± 0.0	(−)	(−)
12		5.0	(−)	(−)	(−)	(−)	(−)	(−)

The biological experiment was carried out in triplicate. Values are given as mean ± standard error.

(G + ve) Gram-Positive, (G − ve) Gram-Negative, (−) Negative.

## Conclusion

The present study not only highlights the role of Zn doping in tailoring structural, optical, and photocatalytic properties but also positions Zn-doped Mn₂O₃ as a formidable ally in environmental cleanup. Zinc-doped Mn_2_O_3_ nanoparticles (NPs) with different Zn content (3%, 5%, and 10%) were synthesized by facile route as the precipitation method. XRD analysis confirmed that the doping of manganese oxide by Zn changed the phase structure from Mn_2_O_3_ (cubic) to ZnMn_2_O_4_ (tetragonal structure), and the reflection peak intensity increases as the Zn doping concentration increases. FESEM images showed that the pure Mn_2_O_3_ has round and cubic-shaped NPs, whereas the doped samples with Zn have tetragonal structure. The energy dispersive x-ray (EDX) analysis confirmed well incorporation of Zn in the Mn_2_O_3_ structure. The TEM images of synthesized nanoparticles showed that the substitution with Zn strongly affects the size and morphology of the prepared NPs, where the particle size decreased with a higher Zn ratio. A red-shifted band energy gaps were detected from 2.26 eV to 1.89 eV with increasing Zn content. The Photoluminescence spectrum (PL) reveals that all the emission peaks are in the visible energy region with energies 1.99, 2.19, 2.66, and 2.91 eV, and their intensities increase with increasing Zn content. In addition, the photocatalytic activity of prepared NPs showed that the Methyl Green (MG) photodegrades of 97.5% when exposed to visible light for 90 min at pH = 7 with a dye concentration of 20 mg/L and a photocatalyst dose of 1.5 g/L for zinc doped Mn_2_O_3_ with 10%. The MG’s photodegradation followed a Pseudo first-order kinetic model. Furthermore, the antibacterial activity of Mn_2_O_3_ doped with Zn was evaluated against harmful microorganisms such as *S. aureus*,* E. faecalis*,* K. pneumoniae*,* P. aeruginosa*,* E. coli*,* and C. albicans* using agar well diffusion assays. The results demonstrated that the Mn_2_O_3_ doped with Zn had antimicrobial responses on all strains and improved by increasing Zn content. Therefore, Mn_2_O_3_ samples that doped with Zn nanoparticles showed promise for combating microbial infections.

## Data Availability

The datasets used and/or analysed during the current study available from the corresponding author on reasonable request.

## Declarations

The datasets used and/or analysed during the current study available from the corresponding author on reasonable request.
